# MicroRNA‐200b is a potential biomarker of the expression of PD‐L1 in patients with lung cancer

**DOI:** 10.1111/1759-7714.13653

**Published:** 2020-09-07

**Authors:** Seigo Katakura, Nobuaki Kobayashi, Hisashi Hashimoto, Chisato Kamimaki, Katsushi Tanaka, Sousuke Kubo, Kentaro Nakashima, Shuhei Teranishi, Saki Manabe, Keisuke Watanabe, Nobuyuki Horita, Yu Hara, Masaki Yamamoto, Makoto Kudo, Hongmei Piao, Takeshi Kaneko

**Affiliations:** ^1^ Department of Pulmonology Yokohama City University Graduate School of Medicine Yokohama Japan; ^2^ Respiratory Disease Center Yokohama City University Medical Center Yokohama Japan; ^3^ Department of Respiratory Medicine Affiliated Hospital of Yanbian University Yanji China

**Keywords:** Biomarkers, exosomes, miRNA, non‐small cell lung cancer, programmed cell death 1 ligand 1

## Abstract

**Background:**

Advanced non‐small cell lung cancer (NSCLC) has a high mortality rate and poor prognosis. However, outcomes have gradually improved after the introduction of novel immunotherapies, including immune checkpoint inhibitors (ICIs). Although programmed death‐ligand 1 (PD‐L1) expression in tumor tissues is a known biomarker for guiding ICI treatment of NSCLC, challenges such as difficulty of liquid biopsy and heterogeneous results during treatment persist. This study evaluated the potential of miR200b as a surrogate biomarker for PD‐L1 expression.

**Methods:**

We used the human lung cancer cell lines H226, H460, H520, A549, and H1975. miR200b expression in blood and bronchoscopy specimens of NSCLC patients was evaluated using reverse‐transcription‐quantitative PCR. Using flow cytometry, PD‐L1 expression in vitro, as well as in tumor tissues, was evaluated after transfection with a mimic miR200b or siRNA.

**Results:**

miR200b expression negatively correlated with PD‐L1 expression in all cell lines. The induction or knockdown of miR200b also altered PD‐L1 expression in vitro. The patient group with a PD‐L1 tumor proportion score ≥ 50% had significantly lower miR200b expression in the bronchoscopy specimens (*P* = 0.025) and serum‐derived exosomes (*P* = 0.022) than that with PD‐L1 tumor proportion score < 50%.

**Conclusions:**

miR200b can regulate PD‐L1 expression in lung cancer cells, and miR200b expression in clinical specimens negatively correlated with PD‐L1 expression. Thus, miR200b may be a useful surrogate biomarker for PD‐L1 expression in lung cancer patients.

**Key points:**

**Significant findings of the study:**

- High PD‐L1 expression was linked to low miR200b expression, whereas low PD‐L1 expression was linked to high miR200b expression in human lung cancer patients. Thus, miR200b overexpression or silencing can control PD‐L1 expression in cancer cells.

**What this study adds**

- We demonstrated the potential of miR200b as a surrogate biomarker for PD‐L1 expression in lung cancer patients.

## Graphical abstract

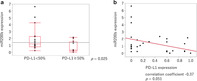

Relative to patients with a PD‐L1 tumor proportion score of <50%, patients with a PD‐L1 tumor proportion score of =50% had significantly lower miR200b expression in the bronchoscopy specimens and serum‐derived exosomes.

## Introduction

The poor prognosis of non‐small cell lung cancer (NSCLC) is largely attributed to the unresectable, advanced status of most patients at the time of diagnosis. In these advanced cases, the administration of therapeutic agents is the mainstay of treatment. Recently, immunotherapy with an immune checkpoint inhibitor (ICI), together with molecular targeted therapy, has improved the prognosis of advanced NSCLC.^1^, ^2^, ^3^, ^4^, ^5^, ^6^, ^7^, ^8^, ^9^

Although ICIs are effective in cases with high tumor mutation burden and high programmed death‐ligand 1 (PD‐L1) expression, the establishment of biomarkers that can more accurately predict treatment outcome is necessary as ICIs potentially have serious adverse events and are costly.^10^ Among biomarkers for predicting treatment outcome, the most established is PD‐L1 expression assessed using the tumor proportion score (TPS) in tumor tissues.^6^, ^7^, ^8^ Nevertheless, a high PD‐L1 TPS (≥50%) has only been associated with a 44.8% response rate to pembrolizumab monotherapy, indicating that the PD‐L1 TPS alone is an insufficient biomarker.^6^ Furthermore, using PD‐L1 expression to guide ICI treatment is limited as PD‐L1 expression can vary according to the biopsy site, even for the same tumor,^11^, ^12^ and changes before and after chemotherapy.^13^, ^14^, ^15^ Finally, an optimal liquid biopsy method has not yet been established.

MicroRNAs (miRNAs) are single‐stranded, small non‐coding RNAs composed of approximately 22 bases and are involved in the post‐transcriptional regulation of gene expression in eukaryotes. Furthermore, miRNAs can be useful biomarkers for various diseases as they are stably present in bodily fluids, including the blood, urine, and saliva.^16^ Several studies have indicated that some miRNAs are related to PD‐L1 expression in cancer patients.^17^, ^18^ Particularly, miR34a, miR200b, miR197, miR513, miR570, and miR138‐5p^19^, ^20^, ^21^, ^22^, ^23^, ^24^ may be potential surrogate biomarkers for PD‐L1 expression, although we focused on miR200b because our preliminary data indicated that only miR200b is negatively correlated with PD‐L1 expression in NSCLC cell lines and clinical specimens from lung cancer patients (data not shown). Low miR200 expression in tumor tissues has been associated with poor differentiation, a greater likelihood of lymph node metastasis, and resistance to docetaxel and gefitinib, highlighting the relationship between low tumor miR200 expression and poor prognosis among NSCLC patients.^25^, ^26^, ^27^ Several reports have described the associations between PD‐L1 expression and various miRNAs. However, to the best of our knowledge, the association between miRNAs and PD‐L1 expression using clinical specimens has not yet been investigated. Therefore, this study evaluated the potential of miR200b as a surrogate biomarker for PD‐L1 expression in lung cancer patients.

## Methods

### Cell lines and cultures

Human lung cancer cell lines H226, H460, H560, and A549 were obtained from the American Type Culture Collection, whereas H1975 was obtained from the Japanese Cancer Research Resources Bank. All cell lines were cultured at 37°C and 5% CO_2_ in RPMI 1640 medium (Sigma‐Aldrich, St. Louis, MO, USA) supplemented with 10% fetal bovine serum (Biowest, Maine‐et‐Loire, France) and 1% Pen/Strep (Gibco, Waltham, MA, USA). All chemical reagents and antibodies were obtained from Cell Signaling Technology (Danvers, MA) unless otherwise stated.

### Western blotting

The cultured cells were lysed using a cell lysis buffer (1:10 dilution) supplemented with protease/phosphatase inhibitor cocktail (1:100). After homogenization, the total protein concentration was quantified using a Nanodrop 2000 system (Thermo Fisher Scientific, Waltham, MA, USA). After 20 μg of protein was heated at 95°C for five minutes, the samples were separated using electrophoresis and 10% sodium dodecyl sulfate‐polyacrylamide gel (BIORAD, Hercules, CA, USA) and subsequently transferred onto polyvinylidene difluoride membranes (Invitrogen, Carlsbad, CA, USA). The membranes were blocked for one hour using 5% nonfat dry milk in Tris‐buffered saline and Tween 20 (1:10) and then probed overnight at 4°C with primary antibodies targeting PD‐L1 (1:1000) and β‐actin (1:1000). The membranes were washed and incubated for one hour at room temperature with HRP‐linked secondary antibodies (1:2000), and the band intensities were quantified using an Image Quant LAS 500 system (GE Healthcare, Chicago, IL, USA).

### Flow cytometry

The cultured cells were harvested using 1.0 mM EDTA and washed twice in ice‐cold phosphate‐buffered saline supplemented with 10% fetal bovine serum. The cells were incubated for 30 minutes at 4°C with APC‐conjugated antibodies targeting human PD‐L1 or APC‐conjugated isotype control antibodies (1:20; BioLegend, San Diego, CA, USA). Flow cytometry was performed using a FACS Celesta system (BD, Franklin Lakes, NJ, USA), and data analysis was performed using the FlowJo software (v10, BD, Franklin Lakes, NJ, USA).

### Immunofluorescence staining

Each cell line was cultured for 48 hours, and the cells were fixed on chamber slides using 4% paraformaldehyde for 10 minutes and permeabilized using 100% ice‐cold methanol for 10 minutes. After blocking the cells with 3% bovine serum albumin for 60 minutes, the slides were stained overnight at 4°C using primary antibodies targeting PD‐L1 (1:200). Next, secondary antibodies targeting rabbit IgG (1:1000) were added for 60 minutes. Staining was performed using the Antifade Reagent with DAPI (Cell Signaling Technology, Danvers, MA), and the cells were observed under a Keyence BZ‐X800 fluorescence microscope (Osaka, Japan).

### Total RNA isolation and reverse‐transcription

Total RNA from the cell lines and bronchial brush cytology specimens was extracted using an RNeasy mini kit (QIAGEN, Hilden, Germany) according to the manufacturer's instructions. Total RNA was also extracted from the conditioned medium and serum‐derived exosomes using a miRNeasy serum/plasma advanced kit (QIAGEN). The purity and the concentration of the extracted total RNA were measured using the Nanodrop 2000 system (Thermo Fisher Scientific). cDNA was reverse‐transcribed using 10 ng of total RNA, a TaqMan MicroRNA Reverse‐Transcription Kit (Applied Biosystems, Foster City, CA, USA), and the primers from TaqMan microRNA assays (Applied Biosystems, Foster City, CA, USA).

### Polymerase chain reaction (PCR)

A previous report has indicated that miR200b is negatively correlated with PD‐L1 expression.^22^ Thus, we performed reverse‐transcription–quantitative PCR (RT‐qPCR) using the reverse‐transcription samples, TaqMan Universal PCR Master Mix (Thermo Fisher Scientific), and the primers from TaqMan microRNA assays (Applied Biosystems; assay ID: 002251) on CFX96 Touch Real‐Time PCR Detection System (BIORAD) according to the manufacturer's protocol. miRNA expression levels were normalized to U6 small nuclear RNA (Applied Biosystems; assay ID: 001973), and the differences in expression were measured relative to the control using the 2^‐ΔΔCq^ method (ΔCq = Cq_target_ − Cq_rereference_).

### Mimicking and silencing miR200b

The cell lines were seeded for 24 hours before transfection with mimic miR200b (GeneDesign, Osaka, Japan), S‐TuD miR200b (GeneDesign) as negative control (Invitrogen), or Stealth™ RNAi siRNAs (Invitrogen). Transfection was performed using Lipofectamine 2000 (final concentration: 10 nM; Invitrogen) according to the manufacturer's protocol and analyzed after 48 hours. The sequences used were as follows: mimic miR200b (upstream primer, 5′‐CAUCUUACUGGGCAGCAUUGGA‐3′) and S‐TuD miR200b (upstream primer, 5′‐GACGGCGCUAGGAUCAUCAACUCCAAUGCUGCCCAGUAAGAUGCAAGUAUUCUGGU‐3′).

### Patients and samples

**Figure 1:**
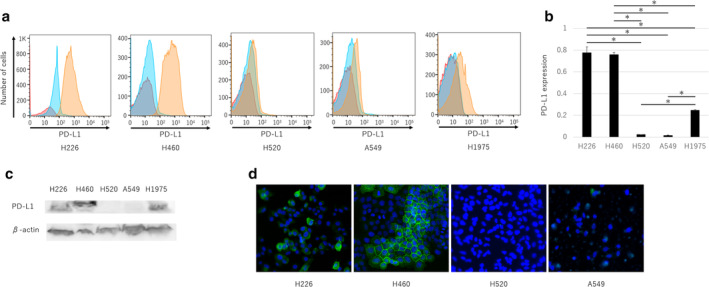
Programmed death‐ligand 1 (PD‐L1) expression and miRNA in human lung cancer cell lines H226, H460, H560, H1975, and A549. (**a**) Flow cytometry was used to evaluate PD‐L1 expression in each cell line (red, unstained; blue, isotype control; orange, APC‐PD‐L1). (**b**) PD‐L1 expression presented as the means ± standard error of the mean (SEM) of three independent flow cytometry assays. (**c**) Western blotting was used to evaluate the expression of PD‐L1 and β‐actin. (**d**) PD‐L1 expression was evaluated using immunofluorescence staining (green, GFP‐PD‐L1; blue, DAPI‐cell nucleus). The *P*‐values were determined using the Mann‐Whitney U test (*P < 0.05).

All clinical procedures in this study were approved by the Yokohama City University Ethics Committee (approval number: A181129001) and conformed with the Declaration of Helsinki. Japanese patients with suspected primary lung cancer were prospectively recruited at the Yokohama City University Hospital and Yokohama City University Medical Center between December 2018 and March 2020. Samples were obtained from patients who provided informed consent for the research use of their bronchoscopy specimens. We selected cases that have been histologically diagnosed as NSCLC and evaluated the miRNA expression of the bronchial brush cytology and serum samples obtained before chemotherapy (Fig 1). Before RNA extraction, the red blood cells were removed from the bronchial brush cytology samples using a RBC lysis buffer (Biolegend, San Diego, CA, USA), and the exosomes were extracted from serum samples using a miRCURY Exosome Serum/Plasma Kit (QIAGEN). The TPS for PD‐L1 expression was evaluated by a pathologist based on the immunohistochemical staining for PD‐L1 (22C3 pharmDx; Dako, Carpinteria, CA, USA).

### Statistical analysis

Data were analyzed using the JMP Pro software (v12, SAS institute, SAS Campus Drive, Cary, NC, USA). Intergroup differences were evaluated using the Mann‐Whitney U test, and intergroup correlations were evaluated using the Pearson's correlation coefficient. Data are presented as the mean ± standard error of the mean, unless otherwise stated, and *P*‐values of <0.05 were considered statistically significant.

## Results

### PD‐L1 expression is different in human lung cancer cell lines

We found high PD‐L1 expression in the H226 and H460 cells and low PD‐L1 expression in the H520, A549, and H1975 cells using flow cytometry (Fig 1a,b) and observed similar results using western blotting (Fig 1c). Fluorescent immunohistochemistry also revealed high PD‐L1 expression in the H226 and H460 cells and low PD‐L1 expression in the H520 and A549 cells (Fig 1d).

### miR200b expression is inversely related to PD‐L1 expression

**Figure 2:**
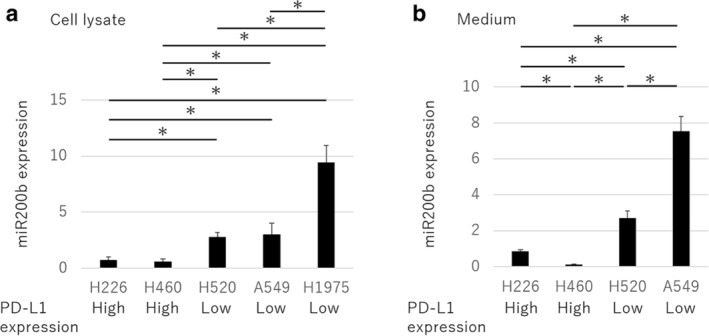
miR200b expression in cell lysates and the conditioned medium of human lung cancer cell lines with high and low PD‐L1 expression. The mean ± SEM of five independent reverse‐transcription‐quantitative PCR (RT‐qPCR) assays of (**a**) cell lysates and (**b**) the conditioned medium of human lung cancer cells. The *P*‐values were determined using the Mann‐Whitney U test (*P < 0.05).

We found low miR200b expression in the H226 and H460 cells and high miR200b expression in the H520, A549, and H1975 cells (Fig 2a). Similar results were obtained when the conditioned medium was used for RNA extraction (Fig 2b). These results indicate that miR200b expression is inversely related to PD‐L1 expression in human lung cancer cells.

### PD‐L1 expression in vitro is regulated by miR200b

**Figure 3:**
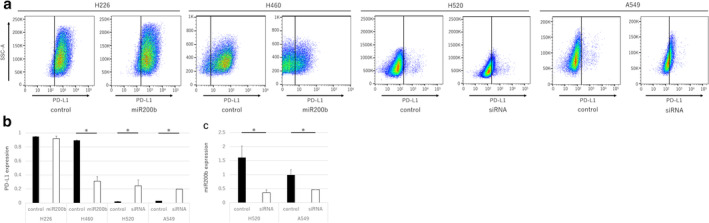
Expression of PD‐L1 and miR200b after transfection with mimic miR200b or miR200b siRNA. (**a**) Flow cytometry was used to evaluate PD‐L1 expression in H226, H460, H520, and A549 cells after transfection with a control miRNA, mimic miR200b, or S‐TuD. (**b**) PD‐L1 expression based on three independent assays. (**c**) miR200b expression in H520 and A549 cells after transfection with the control miRNA and S‐TuD based on three independent RT‐qPCR assays. Data are presented as the mean ± SEM, and the *P*‐values were determined using the Mann‐Whitney U test (*P < 0.05).

PD‐L1 expression was significantly reduced by mimic miR200b in H460 cells (with normally high PD‐L1 expression), but was increased after silencing using S‐TuD miR200b in H520 and A549 (with normally low PD‐L1 expression) cells (Fig 3a‐c, *P* < 0.05). These results indicate that miRNA200b regulates PD‐L1 expression in human lung cancer cells.

### miR200b is inversely related to PD‐L1 expression in specimens from lung cancer patients

**Figure 4:**
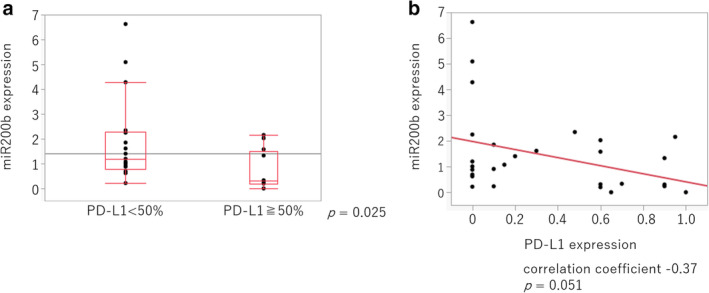
Relationship between miR200b expression and the PD‐L1 tumor proportion score (TPS) in cells from lung cancer patients. (**a**) miR200b expression in human cancer cells collected via bronchoscopy was detected using RT‐qPCR. PD‐L1 expression was assessed immunohistochemically, and the patients were divided into two groups with high (<50%) and low (≥50%) PD‐L1 expression. The *P*‐values were determined using the Mann‐Whitney U test. (**b**) The correlation between miR200b expression and the PD‐L1 TPS was evaluated using Pearson's correlation coefficients.

**Figure 5:**
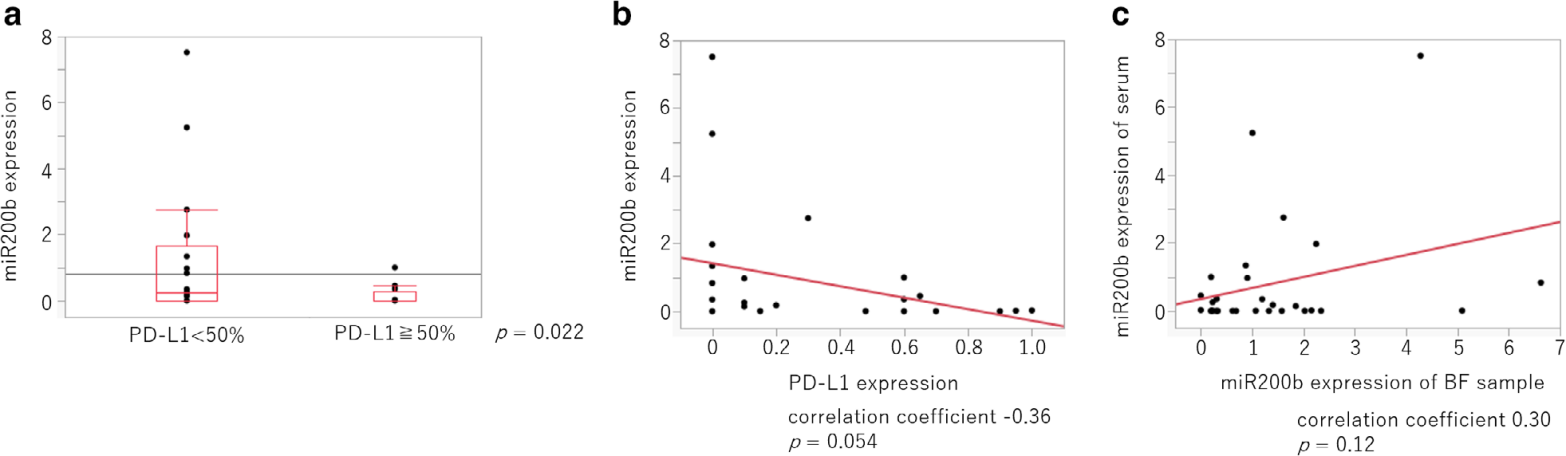
Relationship between miR200b expression in serum‐derived exosomes and the PD‐L1 TPS in cells from lung cancer patients. (**a**) miR200b expression in serum‐derived exosomes was detected using RT‐qPCR, whereas PD‐L1 expression was assessed immunohistochemically (<50% vs. ≥50%). The *P*‐values were determined using the Mann‐Whitney U test. (**b**) The correlation between miR200b expression in serum‐derived exosomes and the PD‐L1 TPS was analyzed using Pearson's correlation coefficients. (**c**) The correlation between miR200b expression in lung cancer tissues and that in serum‐derived exosomes was analyzed using Pearson's correlation coefficients.

Patient characteristics are shown in Table 1, and the immunohistochemical expression of PD‐L1 was categorized as low (<50%) or high (≥50%). We observed that the specimens with high PD‐L1 expression had significantly lower miR200b expression (Fig 4a). There was a weak negative correlation (correlation coefficient: −0.37) between the PD‐L1 TPS and the miR200b expression in lung cancer tissues (Fig 4b). Furthermore, in serum‐derived exosomes, significantly lower miR200b expression was observed in patients with high PD‐L1 expression (Fig 5a), and the miR200b expression exhibited a weak negative correlation (correlation coefficient: −0.36) with PD‐L1 expression (Fig 5b). Further, we investigated the concordance of miR200b expression in lung cancer tissues and serum‐derived exosomes and found a weak positive correlation (correlation coefficient: 0.30) (Fig 5c).

**Table 1:** Patient characteristics

	*n* = 29
Age in years, median (range)	73 (45–86)
Male sex, *n* (%)	19 (65.5%)
Histology, *n* (%)	
Adeno	16 (55.1%)
Squamous	8 (27.6%)
NSCLC	5 (17.2%)
PD‐L1 TPS, *n* (%)	
0%	10 (34.5%)
1–49%	8 (27.6%)
≥50%, n (%)	11 (37.9%)
Driver mutation, *n* (%)	
*EGFR*	5 (17.2%)
*ALK*	2 (7.0%)
Stage, *n* (%)	
I	5 (17.2%)
II	3 (10.3%)
III	10 (34.5%)
IV	11 (37.9%)

Adeno, adenocarcinoma; NSCLC, non‐small cell lung cancer; PD‐L1, programmed death‐ligand 1; Squamous, squamous cell carcinoma; TPS, tumor proportion score.

### Relationship between miR200b and PD‐L1 expression in patients without driver oncogene mutation is also inverse

**Figure 6:**
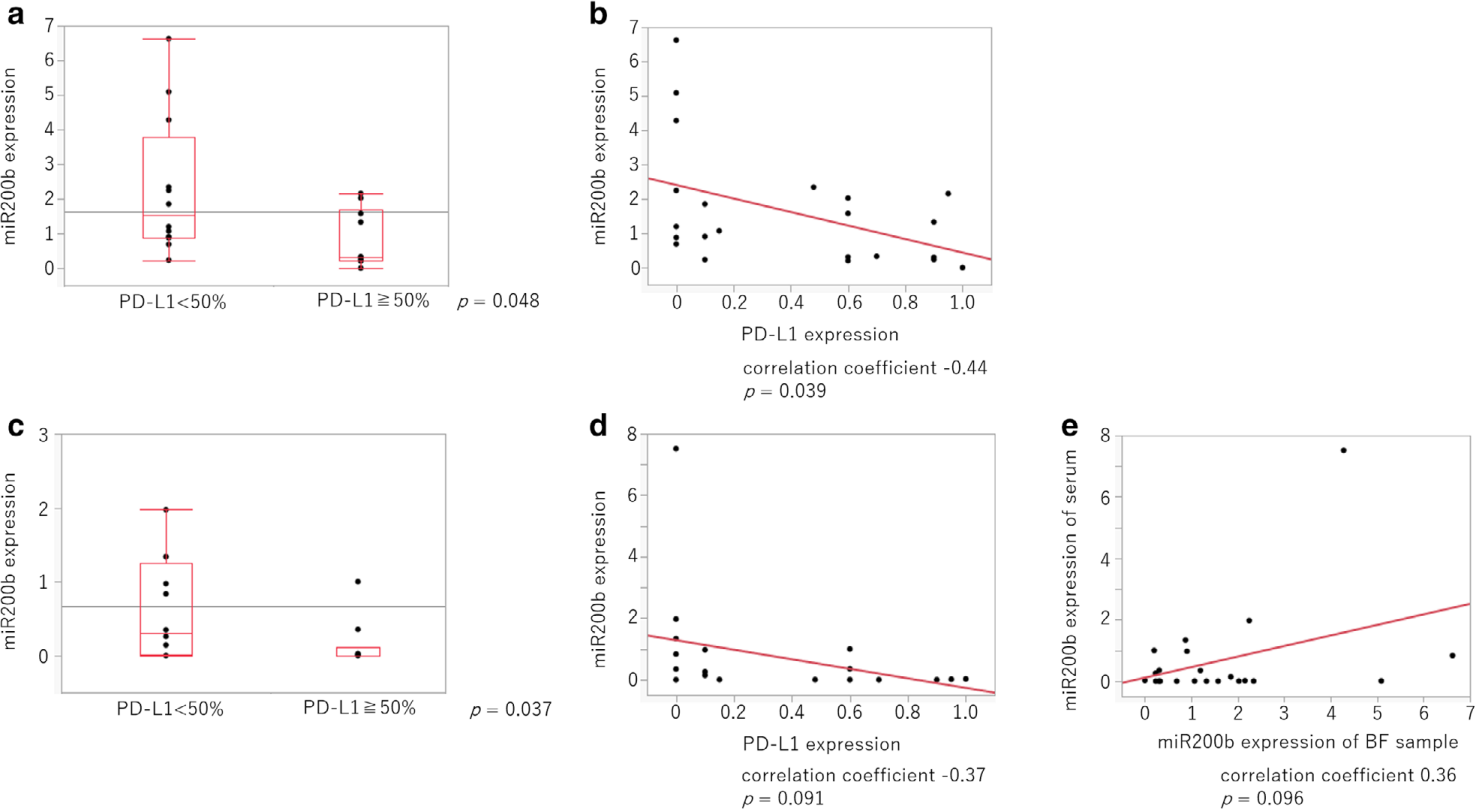
Relationship between miR200b expression and the PD‐L1 TPS in driver mutation‐negative lung cancer patients. (**a**) miR200b expression in human cancer cells collected via bronchoscopy was detected using RT‐qPCR. PD‐L1 expression was assessed immunohistochemically, and the patients were divided into two groups with high (<50%) and low (≥50%) PD‐L1 expression. The *P*‐values were determined using the Mann‐Whitney U test. (**b**) The correlation between miR200b expression and the PD‐L1 TPS was evaluated using Pearson's correlation coefficients. (**c**) miR200b expression in serum‐derived exosomes was detected using RT‐qPCR, whereas PD‐L1 expression was assessed immunohistochemically (<50% vs. ≥50%). (**d**) The correlation between miR200b expression in serum‐derived exosomes and the PD‐L1 TPS is shown. (**e**) The correlation between miR200b expression in lung cancer tissue and miR200b expression in serum‐derived exosomes is shown. The *P*‐values were determined using the Mann‐Whitney U test, and the correlations were analyzed using Pearson's correlation coefficients.

PD‐L1 expression is regulated differently in epidermal growth factor receptor (EGFR) gene wild‐type and mutant cases. Therefore, the relationship between miR200b and PD‐L1 expression in patients without the driver oncogene mutation was analyzed. Similar to the results above, a significantly lower miR200b expression in both lung cancer tissues and serum‐derived exosomes was observed in patients with high PD‐L1 expression (Fig 6a,c), (correlation coefficient: −0.44). Similar results were obtained for each correlation in each sample type (Fig 6b,d,e) (correlation coefficient: −0.37).

## Discussion

This study revealed that miR200b may regulate PD‐L1 expression in cancer cells and that miR200b expression is inversely related to PD‐L1 expression in lung cancer patients. These findings suggest that miR200b might be a useful surrogate marker for PD‐L1 expression in lung cancer patients, which could then be used to predict the clinical efficacy of ICI treatment.

miRNAs have various functions that influence biological processes, such as cell development, differentiation, and proliferation, as well as the maintenance of homeostasis and apoptosis.^28^ Moreover, miRNAs are useful biomarkers for various diseases because they are stable and simple to detect in pathological specimens, blood, urine, and saliva.^16^, ^29^, ^30^ Various applications of miRNAs, such as use as diagnostic and chemoresistance biomarkers and therapeutic targets, have been reported for NSCLC.^31^, ^32^, ^33^ Particularly, the miR200 family, including miR200a, miR200b, miR200c, miR141, and miR429, regulates the epithelial phenotype of cancer cells. In tumors, miR200b is mainly involved in epithelial–mesenchymal transition via the zinc‐finger E‐box binding homeobox 1 and helps control tumor metastasis and progression.^34^

Several studies have indicated that the expression of some miRNAs is related to PD‐L1 expression in cancer patients.^17^, ^18^ For example, PD‐L1 expression is inversely related to miR34a in acute myeloid leukemia, miR200b and miR197 in NSCLC, miR513 in biliary epithelial cells, miR570 in hepatocellular carcinoma, and miR138‐5p in colon cancer.^19^, ^20^, ^21^, ^22^, ^23^, ^24^ Thus, miRNAs can be potential surrogate biomarkers for PD‐L1 expression. A previous report has also indicated that the 3′‐UTR of PD‐L1 contains two sites for possible binding to members of the miR200 family (miR200a, miR200b, and miR200c), and that PD‐L1 expression is directly regulated by the miR200 family based on the results of luciferase reporter assay.^22^

This study revealed that miR200b expression is inversely related to PD‐L1 expression and that miR200b influenced PD‐L1 expression in human lung cancer cells, although the underlying molecular mechanisms remain unclear. Determination of miR200b expression in serum samples might address these limitations as these specimens are simple to obtain and could be collected throughout the course of treatment.

This study has several limitations that merit consideration. First, we only evaluated 29 clinical specimens from two hospitals. Second, we did not evaluate the relationship between the clinical efficacy of ICI treatment and the expression of miR200b before or after ICI treatment because only seven patients had been treated with ICIs. Therefore, additional studies using larger samples are needed to examine the relationship between serum‐derived exosomal expression of miR200b and ICI efficacy, as well as whether exosomal miR200b expression varies before, during, or after ICI treatment.

Taken together, our results indicate an inverse relationship between miR200b and PD‐L1 expression. Further studies are necessary to validate the applicability of miR200b as a biomarker to guide ICI treatment in lung cancer patients.

## Disclosure

The authors declare no conflicts of interest.
